# Identification and Evaluation of Angiogenesis-Related Proteins That Predict Major Adverse Cardiovascular Events in Patients with Peripheral Artery Disease

**DOI:** 10.3390/jcdd11120402

**Published:** 2024-12-13

**Authors:** Ben Li, Farah Shaikh, Houssam Younes, Batool Abuhalimeh, Jason Chin, Khurram Rasheed, Abdelrahman Zamzam, Rawand Abdin, Mohammad Qadura

**Affiliations:** 1 Department of Surgery, University of Toronto, Toronto, ON M5S 1A1, Canada; benx.li@mail.utoronto.ca; 2 Division of Vascular Surgery, St. Michael’s Hospital, Unity Health Toronto, University of Toronto, Toronto, ON M5B 1W8, Canada; farah.shaikh@unityhealth.to (F.S.); abdelrahman.zamzam@unityhealth.to (A.Z.); 3 Institute of Medical Science, University of Toronto, Toronto, ON M5S 1A1, Canada; 4 Temerty Centre for Artificial Intelligence Research and Education in Medicine (T-CAIREM), University of Toronto, Toronto, ON M5S 1A1, Canada; 5 Heart, Vascular, & Thoracic Institute, Cleveland Clinic Abu Dhabi, Abu Dhabi 112412, United Arab Emirates; hkyounesmd@gmail.com (H.Y.); abuhalb@ccf.org (B.A.); jason.a.chin@medstar.net (J.C.); khurram_rasheed@urmc.rochester.edu (K.R.); 6 Department of Medicine, McMaster University, Hamilton, ON L8S 4L8, Canada; rawand.abdin@medportal.ca; 7 Li Ka Shing Knowledge Institute, St. Michael’s Hospital, Unity Health Toronto, University of Toronto, Toronto, ON M5B 1W8, Canada

**Keywords:** angiogenesis, endostatin, ANGPTL4, ANGPTL3, major adverse cardiovascular events, prognosis, peripheral artery disease

## Abstract

Background: The most common cause of death in patients with peripheral artery disease (PAD) are major adverse cardiovascular events (MACEs), including myocardial infarction (MI) and stroke. However, data on biomarkers that could be used to help predict MACEs in patients with PAD to guide clinical decision making is limited. Angiogenesis-related proteins have been demonstrated to play an important role in systemic atherosclerosis and may act as prognostic biomarkers for MACEs in patients with PAD. In this study, we evaluated a large panel of angiogenesis-related proteins and identified specific biomarkers associated with MACEs in patients with PAD. Methods: We conducted a prognostic study using a prospectively recruited cohort of 406 patients (254 with PAD and 152 without PAD). Plasma concentrations of 22 circulating angiogenesis-related proteins were measured at baseline, and the cohort was followed for 2 years. The primary outcome was 2-year MACEs (composite of MI, stroke, or death). Plasma protein concentrations were compared between PAD patients with and without 2-year MACEs using Mann–Whitney U tests. Differentially expressed proteins were further investigated in terms of their prognostic potential. Specifically, Cox proportional hazards analysis was performed to determine the independent association between differentially expressed proteins and 2-year MACEs, controlling for all baseline demographic and clinical characteristics, including existing coronary artery disease and cerebrovascular disease. Kaplan–Meier analysis was conducted to assess 2-year freedom from MACEs in patients with low vs. high levels of the differentially expressed proteins based on median plasma concentrations. Results: The mean age of the cohort was 68.8 (SD 11.1), and 134 (33%) patients were female. Two-year MACEs occurred in 63 (16%) individuals. The following proteins were significantly elevated in PAD patients with 2-year MACEs compared to those without 2-year MACEs: endostatin (69.15 [SD 58.15] vs. 51.34 [SD 29.07] pg/mL, *p* < 0.001), angiopoietin-like protein 4 (ANGPTL4) (0.20 [SD 0.09] vs. 0.12 [SD 0.04] pg/mL, *p* < 0.001), and ANGPTL3 (51.57 [SD 21.92] vs. 45.16 [SD 21.90] pg/mL, *p* = 0.001). Cox proportional hazards analysis demonstrated that these three proteins were independently associated with 2-year MACEs after adjusting for all baseline demographic and clinical characteristics: endostatin (HR 1.39 [95% CI 1.12–1.71] *p* < 0.001), ANGPTL4 (HR 1.35 [95% CI 1.08–1.68], *p* < 0.001), and ANGPTL3 (HR 1.35 [95% CI 1.12–1.63], *p* < 0.001). Over a 2-year follow-up period, patients with higher levels of endostatin, ANGPTL4, and ANGPTL3 had a lower freedom from MACEs. Supplementary analysis demonstrated that these three proteins were not significantly associated with 2-year MACEs in patients without PAD. Conclusions: Among a panel of 22 angiogenesis-related proteins, endostatin, ANGPTL4, and ANGPTL3 were identified to be independently and specifically associated with 2-year MACEs in patients with PAD. Measurement of plasma concentrations of these proteins can support MACE risk stratification in patients with PAD, thereby informing clinical decisions on multidisciplinary referrals to cardiologists, neurologists, and vascular medicine specialists and guiding aggressiveness of medical treatment, thereby improving cardiovascular outcomes in patients with PAD.

## 1. Introduction

Peripheral artery disease (PAD) involves atherosclerosis of the lower extremity arteries and affects over 200 million people globally [1]. While there is a strong link between PAD and major adverse limb events that can result in amputation, the primary causes of death in PAD patients are major adverse cardiovascular events (MACEs), such as myocardial infarction (MI) and stroke [2]. This is because there is a significant association between PAD and coronary artery disease (CAD) and cerebrovascular disease (CVD) [3]. This association can be attributed to systemic atherosclerosis, which is influenced by common risk factors, including older age, hypertension, diabetes, dyslipidemia, smoking, and genetic factors, among others [4]. Precision medicine has become an important consideration for the diagnosis and management of various diseases [5]. Consequently, it is crucial to identify patients with PAD who are at high risk for MACEs to improve precision in the diagnosis and treatment of this condition [5]. Ultimately, these patients can receive multidisciplinary evaluations that would aid in cardiovascular disease risk modification/reduction [6]. One approach to addressing this issue is to identify novel biomarkers that can predict MACEs in patients with PAD. Our group has previously identified several biomarkers that can predict adverse limb events in patients with PAD, including fatty acid binding proteins [7,8,9,10,11], inflammatory proteins [12], and Cystatin C [13]; however, the investigation of biomarkers that can predict MACEs in patients with PAD has been limited.

Angiogenesis involves the formation of novel blood vessels, often in response to vascular insults, such as ischemia, in the setting of atherosclerosis [14]. Angiogenesis-related proteins such as angiopoietin-like protein 4 (ANGPTL4), ANGPTL3, and endostatin have been identified in multiple pathologies, including PAD, CAD, and CVD [15,16,17]. In fact, over 20 angiogenesis-related proteins have been demonstrated to be associated with PAD, CAD, and CVD [18,19,20,21,22]. Therefore, angiogenesis-related proteins may act as biomarkers for the early identification of MACEs in patients with PAD [14]. The selection of these 22 angiogenesis-related proteins for analysis in this study is grounded in their extensive research and strong correlations with cardiovascular diseases, highlighting their potential relevance in predicting MACEs in patients with PAD [18,19,20,21,22]. While previous studies have demonstrated links between these proteins and cardiovascular conditions, few have specifically investigated their prognostic implications for MACEs in PAD patients. The aim of this study is to identify prognostic biomarkers that can predict MACEs in individuals with PAD using a large panel of angiogenesis-related proteins, with the goal of identifying high-risk patients who may benefit from more aggressive medical management for systemic atherosclerosis to prevent adverse events.

## 2. Materials and Methods

### 2.1. Ethics Approval

This study was approved by the research ethics board at Unity Health Toronto, University of Toronto, Canada, on 8 February 2017 (REB # 16-365). Prior to participation, all individuals provided informed consent, and all procedures adhered strictly to the principles outlined in the Declaration of Helsinki [23].

### 2.2. Design

This was a prognostic study, and the findings were reported in accordance with the Transparent Reporting of a Multivariable Prediction Model for Individual Prognosis or the Diagnosis + Artificial Intelligence (TRIPOD + AI) statement [24].

### 2.3. Patient Recruitment

This study involved the prospective recruitment of patients, both with and without PAD, who sought care at ambulatory clinics within our institution from January 2018 to August 2019 at Unity Health Toronto, University of Toronto, Canada. PAD was diagnosed based on an Ankle–Brachial Index (ABI) of less than 0.9 or a Toe–Brachial Index (TBI) of less than 0.7 along with absent or diminished pedal pulses [25]. Conversely, non-PAD was defined by an ABI of 0.9 or higher, a TBI of 0.7 or higher, and normal pedal pulses [25]. Exclusion criteria included patients with acute limb ischemia, acute coronary syndrome, or elevated troponin levels within the past three months.

### 2.4. Baseline Characteristics

Baseline characteristics recorded in this study included age, sex, hypertension (defined as systolic blood pressure ≥ 130 mmHg, diastolic blood pressure ≥ 80 mmHg, or currently on blood pressure-lowering therapy [26,27]), dyslipidemia (total cholesterol > 5.2 mmol/L, triglycerides > 1.7 mmol/L, or currently on lipid-lowering therapy [26,27]), diabetes (hemoglobin A1c ≥ 6.5% or currently on antidiabetic medication [26,27]), current and past smoking habits, and the presence of CAD, congestive heart failure (CHF), and a history of stroke. Definitions for cardiovascular risk factors were based on the guidelines established by the American College of Cardiology [26,27].

### 2.5. Quantification of Plasma Protein Levels

Blood samples were collected from patients, and the plasma concentrations of 22 angiogenesis-related proteins were measured in duplicate using a commercially available LUMINEX assay (Bio-Techne, Minneapolis, MN, USA), in accordance with the manufacturer’s instructions [28]. The LUMINEX assay has a catalog number of LUHM000, and the intended purpose of the assay is to measure a broad range of plasma protein concentrations in human samples [28]. This assay is approved for clinical evaluation by Clinical Laboratory Improvement Amendments (CLIAs) [28]. The following proteins were chosen based on their involvement in various metabolic processes associated with angiogenesis and important associations with cardiovascular diseases: endostatin, ANGPTL4, ANGPTL3, tumor necrosis factor receptor I (TNFRI), a disintegrin-like metalloproteinase with thrombospondin motif type 1 member 13 (ADAMTS13), ANGPTL6, endothelial cell-specific molecule-1 (ESM-1), thyroid peroxidase (Tpo), Skp, Cullin, F-box containing complex (SCF), CXC motif chemokine ligand 1 (CXCL1), tumor necrosis factor inducible gene 14 (TSG-14), pre-B cell colony-enhancing factor (PBEF)/Visfatin, intercellular adhesion molecule-1 (ICAM-1), urokinase-type plasminogen activator receptor (uPAR), bone morphogenetic protein 2 (BMP-2), angiopoietin-1, furin, Proprotein Convertase Subtilisin/Kexin Type 9 (PCSK9), kidney injury molecule-1 (KIM-1), endoglin/cluster of differentiation 105 (CD105), P-selectin (CD62P), and Tie-2. The analysis of multiple angiogenesis-related proteins aims to identify novel biomarkers for PAD. Before sample analysis, Fluidics Verification and Calibration bead kits (Luminex Corp, Austin, TX, USA) [29] were used to calibrate the MagPix analyzer (Luminex Corp, Austin, TX, USA) [30]. To minimize inter-assay variability, all sample analyses were performed on the same day. The intra-assay and inter-assay coefficients of variability for the samples were both less than 10%. Given the low intra-assay and inter-assay coefficients of variability (<10%), samples were analyzed in duplicates rather than triplicates. A minimum of 50 beads for each protein were collected and analyzed using Luminex xPonent software version 4.3 (Luminex Corp, Austin, TX, USA) [31].

### 2.6. Follow-Up and Outcomes

Outpatient clinic visits were conducted at 1 year and 2 years following the baseline assessment. The primary outcome of interest was 2-year major adverse cardiovascular events (MACEs), which included a composite of stroke, myocardial infarction, and death. Myocardial infarction was defined as acute myocardial ischemia indicated by clinical symptoms, electrocardiogram changes, and troponin elevation. Stroke was defined as the presence of ipsilateral or contralateral neurological deficits lasting more than 24 h. Death was classified as all-cause mortality.

### 2.7. Statistical Analysis

The demographic and clinical characteristics of our cohort were summarized using means and standard deviations (SDs) or counts and proportions. Baseline differences between groups were assessed using independent *t*-tests for continuous variables and chi-square tests for categorical variables. Event rates at 2 years were compared between PAD and non-PAD patients using chi-square tests. Plasma protein concentrations were analyzed between PAD patients with and without 2-year MACEs using Mann–Whitney U tests. Proteins that showed differential expression in PAD patients with 2-year MACEs were further analyzed to determine their prognostic potential. Specifically, associations between these proteins and 2-year MACEs were evaluated using Cox proportional hazards analysis, adjusting for age, sex, hypertension, dyslipidemia, diabetes, past/current smoking, CHF, CAD, and previous stroke. The cohort was stratified into low and high levels of the significant proteins based on median plasma concentrations. Freedom from MACEs over the 2-year period was analyzed using Kaplan–Meier curves, with comparisons made via Cox proportional hazards analysis, and was also adjusted for all baseline characteristics. This stratified analysis aimed to elucidate the clinical prognostic value of each significant protein, helping clinicians understand how a patient’s risk trajectory for MACEs differs based on low versus high protein levels. Statistical significance was set at a two-tailed *p* < 0.05. All analyses were conducted using SPSS software version 23 (SPSS Inc., Chicago, IL, USA) [32].

## 3. Results

### 3.1. Patients

In total, 406 patients were included in this study (254 with PAD and 152 without PAD). Patients with PAD were older (mean age 71 [SD 10] vs. 65 [SD 13] years, *p* < 0.001) and exhibited higher rates of hypertension (85% vs. 59%, *p* < 0.001), dyslipidemia (82% vs. 66%, *p* < 0.001), diabetes (47% vs. 18%, *p* < 0.001), CAD (39% vs. 24%, *p* = 0.002), and a history of stroke (20% vs. 11%, *p* = 0.035). Additionally, a higher proportion of PAD patients were past or current smokers (82% vs. 60%, *p* < 0.001) (Table 1).

### 3.2. Major Adverse Cardiovascular Events

During the 2-year follow-up period, MACEs occurred in sixty-three individuals (16%), with the following distribution: MI in fifty-one patients (13%), stroke in seventeen patients (4%), and death in five patients (1%). There were no significant differences in 2-year event rates between patients with PAD and those without PAD (Table 2).

### 3.3. Plasma Concentrations of Angiogenesis-Related Proteins

Among the twenty-two angiogenesis-related proteins analyzed, three were significantly elevated in PAD patients who experienced 2-year MACEs compared to those who did not: endostatin (69.15 [SD 58.15] vs. 51.34 [SD 29.07] pg/mL, *p* < 0.001), ANGPTL4 (0.20 [SD 0.09] vs. 0.12 [SD 0.04] pg/mL, *p* < 0.001), and ANGPTL3 (51.57 [SD 21.92] vs. 45.16 [SD 21.90] pg/mL, *p* = 0.001) (Table 3). Given the elevated plasma concentrations of endostatin, ANGPTL4, and ANGPTL3 in PAD patients who developed 2-year MACEs, their prognostic potential was further investigated in this study. TNFRI was not additionally investigated in this study as it did not reach statistical significance (*p* < 0.05) in terms of its difference in plasma concentration between patients with vs. without MACEs. However, given its previously demonstrated role in cardiovascular diseases [33], future studies with larger sample sizes and sufficient power are needed to investigate the association between TNFRI and MACEs in patients with PAD.

### 3.4. Associations Between Angiogenesis-Related Proteins and Major Adverse Cardiovascular Events

Plasma concentrations of all three angiogenesis-related proteins were independently associated with 2-year MACEs in patients with PAD, even after adjusting for all baseline demographic and clinical characteristics. The hazard ratios were as follows: endostatin (HR 1.39 [95% CI 1.12–1.71], *p* < 0.001), ANGPTL4 (HR 1.35 [95% CI 1.08–1.68], *p* < 0.001), and ANGPTL3 (HR 1.35 [95% CI 1.12–1.63], *p* < 0.001) (Table 4). Additional analysis indicated no significant associations between these proteins and 2-year MACEs in patients without PAD (Table 5). This underscores that endostatin, ANGPTL4, and ANGPTL3 specifically predict 2-year MACEs in patients with PAD.

### 3.5. Kaplan–Meier Analysis

**Figure 1:**
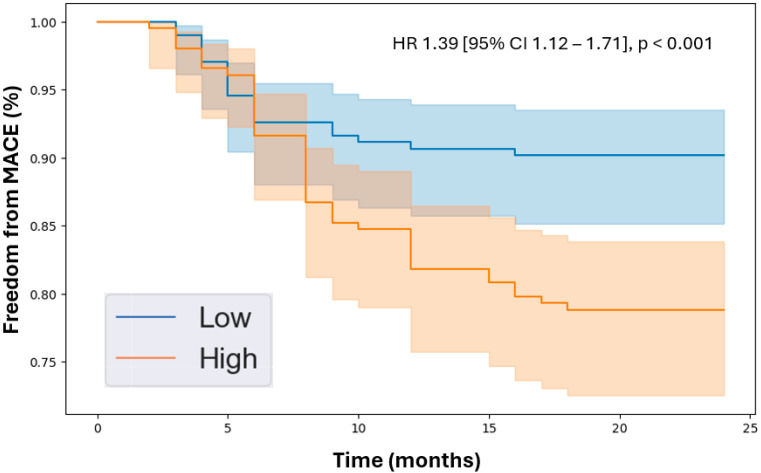
Kaplan–Meier analysis of freedom from major adverse cardiovascular events in patients with peripheral artery disease stratified by low vs. high levels of endostatin based on the median plasma concentration in the cohort (54.6 pg/mL). Cox proportional hazards analysis adjusted for age, sex, hypertension, dyslipidemia, diabetes, past/current smoking, congestive heart failure, coronary artery disease, and previous stroke. Abbreviations: MACE (major adverse cardiovascular event), HR (hazard ratio), CI (confidence interval).

**Figure 2:**
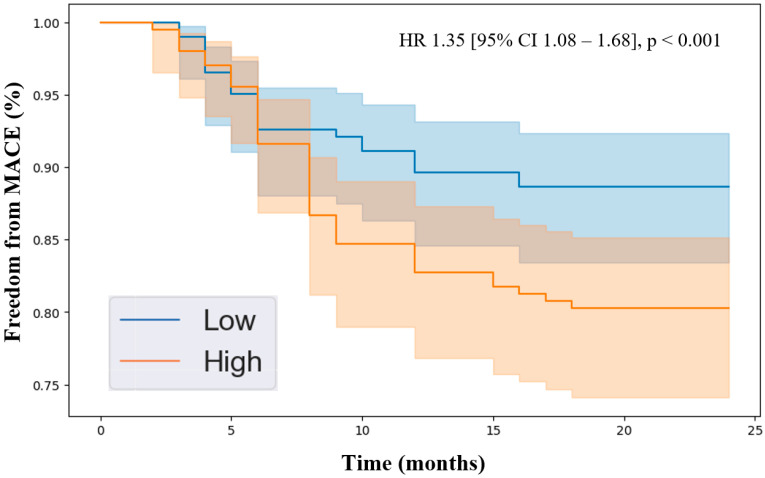
Kaplan–Meier analysis of freedom from major adverse cardiovascular events in patients with peripheral artery disease stratified by low vs. high levels of angiopoietin-like protein 4 (ANGPTL4) based on the median plasma concentration in the cohort (0.19 pg/mL). Cox proportional hazards analysis adjusted for age, sex, hypertension, dyslipidemia, diabetes, past/current smoking, congestive heart failure, coronary artery disease, and previous stroke. Abbreviations: MACE (major adverse cardiovascular event), HR (hazard ratio), CI (confidence interval).

**Figure 3:**
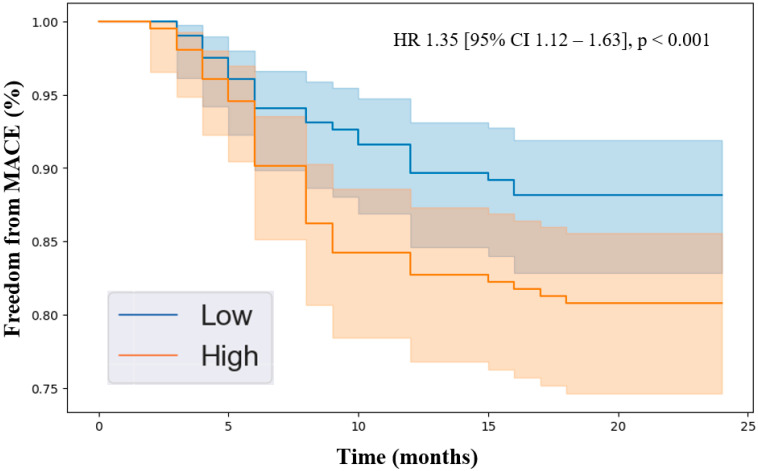
Kaplan–Meier analysis of freedom from major adverse cardiovascular events in patients with peripheral artery disease stratified by low vs. high levels of angiopoietin-like protein 3 (ANGPTL3) based on the median plasma concentration in the cohort (50.4 pg/mL). Cox proportional hazards analysis adjusted for age, sex, hypertension, dyslipidemia, diabetes, past/current smoking, congestive heart failure, coronary artery disease, and previous stroke. Abbreviations: MACE (major adverse cardiovascular event), HR (hazard ratio), CI (confidence interval).

Kaplan–Meier analysis revealed that patients with higher levels of the identified proteins experienced lower freedom from MACEs over the 2-year follow-up period. Specifically, the hazard ratios were endostatin (HR 1.39 [95% CI 1.12–1.71], *p* < 0.001, Figure 1), ANGPTL4 (HR 1.35 [95% CI 1.08–1.68], *p* < 0.001, Figure 2), and ANGPTL3 (HR 1.35 [95% CI 1.12–1.63], *p* < 0.001, Figure 3). Importantly, the Kaplan–Meier analysis performed on the three biomarkers is an estimate of survival probability for MACEs, and, therefore, the data in this study should be carefully interpreted. Future validation studies are needed to confirm the findings.

## 4. Discussion

### 4.1. Summary of Findings

In this study, we identified ANGPTL4, ANGPTL3, and endostatin as angiogenesis-related proteins that are independently associated with 2-year MACEs in patients with PAD, potentially serving as prognostic biomarkers. Several key findings emerged from our analysis. First, among the 22 angiogenesis-related proteins analyzed, ANGPTL4, ANGPTL3, and endostatin were the only ones that were significantly elevated in PAD patients who experienced 2-year MACEs compared to those who did not. Second, we demonstrated independent associations between these proteins and 2-year MACEs in PAD patients, even after controlling for baseline demographic and clinical characteristics, including existing CAD and CVD. Notably, we found no significant associations between these proteins and 2-year MACEs in patients without PAD, indicating that they specifically predict MACEs in the PAD population. Third, by stratifying patients based on median plasma concentrations of ANGPTL4, ANGPTL3, and endostatin, we used Kaplan–Meier analysis to show that those with higher levels of each protein were more likely to develop MACEs over the 2-year follow-up period. This highlights the clinical relevance of these proteins in helping clinicians assess the future MACE risk for their PAD patients. This can help clinicians identify high-risk patients and ensure that they receive appropriate cardiovascular risk-reduction treatment strategies. Overall, the significance of ANGPTL4, ANGPTL3, and endostatin in predicting MACEs in PAD patients underscores the need for further basic science and translational research. This future work should aim to elucidate the biological relationships between these proteins and the development and progression of cardiovascular disease in PAD patients, ultimately guiding the development of targeted therapeutic strategies.

### 4.2. Comparison to the Existing Literature

Aryal and colleagues (2019) reviewed the role of ANGPTL4 in cardiovascular disease and highlighted accumulating evidence showing the direct association between ANGPTL4 and systemic atherosclerosis [15]. Specifically, Muendlein et al. (2014) showed that plasma ANGPTL4 levels significantly predict cardiovascular events in patients with CAD independently of conventional cardiovascular risk factors [34]. Similarly, Dewey and colleagues (2017) showed that genetic and therapeutic antagonism of ANGPTL3 in humans was associated with decreased odds of atherosclerotic cardiovascular disease [35]. Elsewhere, Kim et al. (2024) showed that higher circulating endostatin levels were associated with a greater risk of cardiovascular events in patients undergoing hemodialysis [36]. Our findings corroborate the literature regarding the important roles of ANGPTL4, ANGPTL3, and endostatin in cardiovascular diseases. We additionally demonstrate the prognostic value of these proteins in predicting MACEs, specifically in patients with PAD, which has not been previously investigated. Li et al. (2023) previously demonstrated that angiogenesis-related proteins are associated with major adverse limb events in patients with PAD [18]. In this study, by further demonstrating that angiogenesis-related proteins can predict MACEs in patients with PAD, we additionally show the importance of angiogenesis-related proteins in biological pathways related to the progression of systemic cardiovascular diseases. Therefore, our work highlights the importance of further investigating the mechanistic relationships between angiogenesis-related proteins and the combination of PAD, CAD, and CVD, with the goal of strengthening our understanding of these pathologies and potentially unveiling novel therapeutic strategies.

### 4.3. Explanation of Findings

There are several potential explanations for our findings. First, ANGPTL4 is a protein secreted by numerous cells, including adipocytes, hepatocytes, cardiomyocytes, and macrophages, and it is involved in modulating triacylglycerol homeostasis [37]. ANGPTL4 also stimulates intracellular adipocyte lipolysis through cyclic adenosine monophosphate-dependent protein kinase A signaling [37]. Through these mechanisms, ANGPTL4 is involved in different aspects of angiogenesis, vascular permeability, and lipid metabolism [38]. Folsom and colleagues (2008) showed through the Atherosclerosis Risk in Communities Study (ARIC) that ANGPTL4 has important associations with CAD, CVD, and PAD [39]. Gagnon and colleagues (2024) demonstrated that the inhibition of ANGPTL4 reduced the risk of CAD and diabetes, demonstrating that ANGPTL4 inhibition may represent an effective target for the prevention or treatment of cardiometabolic diseases [40]. The molecular mechanisms through which ANGPTL4 is associated with cardiovascular diseases have been previously described by Aryal and colleagues (2019) [15]. Specifically, ANGPTL4 binds to lipoprotein lipase (LPL) and inhibits its lipolytic activity, resulting in a decrease in the hydrolysis of triglycerides in metabolic tissues, including the heart and muscles [15]. The loss of ANGPTL4 in these tissues increases LPL activity and accelerates the catabolism of triglyceride-rich lipoproteins, reducing circulating triglycerides and low-density lipoprotein–cholesterol (LDL-C), which protects against the progression of atherosclerosis [15]. Therefore, high ANGPTL4 levels may contribute to atherosclerosis development and progression [15]. These studies demonstrate the important role of ANGPTL4 in cardiovascular diseases and may explain why this protein was independently associated with MACEs in our cohort of PAD patients. Second, ANGPTL3 is in the same protein family as ANGPTL4 [41]. Both proteins have a similar domain architecture; after the signal peptide, they have an intrinsically disordered region followed by a coiled-coil domain and a C-terminal fibrinogen-like domain [41]. ANGPTL3 is primarily expressed and secreted by the liver and recently emerged as a key player in lipid metabolism [42]. Specifically, loss-of-function mutations in ANGPTL3 have been demonstrated to result in familial combined hypolipidemia, characterized by low plasma triglycerides, low-density lipoprotein (LDL), and high-density lipoprotein [42]. This lipid-driven mechanism may explain the role of ANGPTL3 in systemic atherosclerosis [42]. Sun and colleagues (2021) showed that circulating levels of ANGPTL3 and ANGPTL4 were independent risk factors for coronary atherosclerosis [43]. Similarly, Hsiao and colleagues (2021) showed that serum ANGPTL3 is associated with peripheral arterial stiffness in patients with CAD [44]. The molecular mechanisms through which ANGPTL3 impacts cardiovascular disease outcomes have been previously described by Luo and colleagues (2023) [16]. Specifically, genetic epidemiological studies have demonstrated that ANGPTL3 loss of function is strongly associated with the lowering of circulating LDL-C, triglyceride-rich lipoproteins, and concurrent risk reduction in the development of CAD [16]. Like ANGPTL4, ANGPTL3 inhibits LPL, and, therefore, the inactivation of ANGPTL3 leads to increased LPL activity and an increased concentration of free fatty acids from triglycerides for uptake and utilization by oxidative tissues [16]. The pharmacological inhibition of ANGPTL3 with monoclonal antibodies shows their safety and efficacy in lowering both LDL-C and triglycerides, thus slowing atherosclerosis progression [16]. Therefore, high levels of ANGPTL3 may be associated with atherosclerosis progression and adverse cardiovascular events, including MI and stroke [16]. The relationship between ANGPTL3 and both PAD and CAD may explain why it predicted MACEs in patients with PAD. Third, endostatin is a 20-kDa C-terminal fragment of type VIII collagen, acting as one of the most potent inhibitors of angiogenesis [45]. It binds various receptors, including vascular endothelial growth factor (VEGF) receptors 2 and 3, members of the integrin family, glypican-1, and glypican-4 [45]. Through these mechanisms, endostatin inhibits angiogenesis, prevents VEGF-C-induced signalling, and downregulates LDL retention and atheroma formation [45]. These mechanisms may explain endostatin’s role in systemic atherosclerosis [45]. Golledge et al. (2014) showed that serum endostatin concentrations are higher in patients with claudication, highlighting its role in PAD [46]. Similarly, Isaacs-Trepanier and colleagues (2020) demonstrated that endostatin mediates an association between endothelial function and cognitive performance in patients with CAD at risk for vascular cognitive impairment [47]. The molecular mechanisms through which endostatin is associated with cardiovascular diseases have been previously described by Li and colleagues (2021) [48]. Specifically, endostatin binds to integrins α5β1 and αV (αVβ3/αVβ5) to inhibit endothelial cell proliferation, migration, and angiogenesis [48]. Endostatin can further increase its anti-angiogenic activity by binding to heparan sulfate proteoglycan and by inhibiting the activity of VEGF by binding to VEGF receptors 1, 2, and 3 [48]. Therefore, high endostatin levels may inhibit angiogenesis, thereby contributing to tissue ischemia and adverse cardiovascular events, including MI and stroke [48]. These studies highlight the important role of endostatin in PAD, CAD, and CVD, thereby potentially explaining why it predicted MACEs in patients with PAD. Taken together, these studies explain the potential mechanisms through which ANGPTL4, ANGPTL3, and endostatin predict MACEs in patients with PAD.

### 4.4. Implications

Our study offers practical implications for guiding clinical decision making in patients with PAD. The measurement of ANGPTL4, ANGPTL3, and endostatin can effectively determine a PAD patient’s risk of MACEs, which is particularly valuable in family practice settings. General practitioners can integrate our findings by measuring plasma levels of ANGPTL4, ANGPTL3, and endostatin to assess MACE risk in PAD patients [49]. Those identified as high risk can be referred for further multidisciplinary evaluation by cardiologists, neurologists, and vascular specialists [50]. In contrast, patients categorized as low risk can continue receiving care from their family physician, focusing on optimizing risk factors through management strategies such as acetylsalicylic acid (ASA), statins, and lifestyle modifications [51]. Once a referral has been made, specialists can use the results of this study to determine how aggressively to treat patients. For example, using our findings to predict a patient’s risk of MACEs over a 2-year period through plasma concentrations of ANGPTL4, ANGPTL3, and endostatin, specific treatment regimens can be determined. Specifically, the addition of low-dose rivaroxaban to ASA has been demonstrated to improve cardiovascular outcomes in patients with stable PAD or CAD [52]. Additionally, angiographic assessment of the coronary and cerebrovascular arteries in high-risk patients may facilitate the early identification of hemodynamically significant atherosclerotic plaques that should be intervened on through endovascular or open interventions [53]. Overall, our research has the potential to enhance care for PAD patients in both generalist and specialist settings by facilitating the early identification of high-risk individuals, guiding the referral process, and supporting clinical decision making regarding the aggressiveness of treatment. This approach can help reduce unnecessary specialist referrals, improve cardiovascular outcomes, and lower healthcare costs [54].

### 4.5. Limitations

Our study has several limitations. Firstly, it was conducted at a single center, necessitating further validation across multiple institutions to assess the generalizability of our findings. Secondly, the outcomes reported were based on a 2-year follow-up period; longer-term follow up is essential to fully understand the prognostic value of the proteins, particularly given the chronic nature of PAD, CAD, and CVD. Thirdly, plasma angiopoietin-1 and -2 levels were not measured in this study. Given their potential roles in vascular function and pathophysiology [55,56], it will be important for future studies to assess their correlations with MACEs in patients with PAD. Additionally, other anti-angiogenic proteins, such as tumstatin and arresten, were not investigated in this study, and it would be prudent for future studies to assess the associations between these proteins and MACEs in patients with PAD [57,58]. Fourthly, drug treatments were not captured in this study. Given the potential associations between some drugs, such as statins, and angiogenesis [59], future studies assessing the interactions between commonly prescribed drugs and ANGPTL4, ANGPTL3, and endostatin would be helpful to further determine the potential value of these biomarkers for cardiovascular prognosis in patients with PAD. Fifthly, the measurement of plasma ANGPTL4, ANGPTL3, and endostatin levels is primarily utilized in research settings. Additional translational research and implementation science are needed to demonstrate the clinical utility and feasibility of integrating these protein measurements into routine care for PAD patients.

## 5. Conclusions

In this study, we identified ANGPTL4, ANGPTL3, and endostatin as circulating angiogenesis-related proteins that are independently associated with 2-year MACEs in patients with PAD, thereby acting as potential biomarkers for systemic atherosclerosis. Importantly, these proteins predicted MACE risk in patients with PAD but not in patients without PAD, highlighting the specificity of these biomarkers for the PAD population. Our findings hold promise for improving MACE risk stratification in patients with PAD, facilitating targeted cardiovascular risk reduction strategies. High-risk patients can be referred for multidisciplinary evaluation by cardiologists, neurologists, and vascular specialists, allowing for more aggressive medical interventions. This is crucial, as most patients with PAD ultimately face mortality from MIs and strokes. Moreover, our results highlight the need for further basic and translational research to investigate the mechanistic relationships between ANGPTL4, ANGPTL3, and endostatin and the development and progression of systemic atherosclerosis. This research may enhance our understanding of the underlying pathogenesis of PAD, CAD, and CVD, thereby informing targeted therapeutic strategies.

## Figures and Tables

**Table 1:** Baseline demographic and clinical characteristics of patients with and without peripheral artery disease.

		Non-PAD (n = 152)	PAD (n = 254)	*p*-Value
Age, years, mean (SD)		65 (13)	71 (10)	<0.001
Sex, n (%)	Male	97 (64)	175 (69)	0.344
	Female	55 (36)	79 (31)	
Hypertension, n (%)		90 (59)	215 (85)	<0.001
Dyslipidemia, n (%)		100 (66)	209 (82)	<0.001
Diabetes, n (%)		27 (18)	120 (47)	<0.001
Smoking, n (%)	Past	64 (42)	147 (58)	<0.001
	Current	28 (18)	60 (24)	
Congestive heart failure, n (%)		2 (1)	12 (5)	0.124
Coronary artery disease, n (%)		36 (24)	99 (39)	0.002
Previous stroke, n (%)		16 (11)	50 (20)	0.035

Abbreviations: PAD (peripheral artery disease), SD (standard deviation).

**Table 2:** Major adverse cardiovascular events over 2 years in patients with and without peripheral artery disease.

Event, n (%)	Overall (n = 406)	Non-PAD (n = 152)	PAD (n = 254)	*p*-Value
Major adverse cardiovascular event	63 (16)	17 (11)	46 (18)	0.085
Myocardial infarction	51 (13)	13 (9)	38 (15)	0.083
Stroke	17 (4)	5 (3)	12 (5)	0.658
Death	5 (1)	2 (1)	3 (1)	0.902

Abbreviation: PAD (peripheral artery disease).

**Table 3:** Plasma concentrations of angiogenesis-related proteins in patients with peripheral artery disease with and without major adverse cardiovascular events.

	No MACE(n = 208)	MACE(n = 46)	*p*-Value
Endostatin	51.34 (29.07)	69.15 (58.15)	<0.001
ANGPTL4	0.12 (0.04)	0.20 (0.09)	<0.001
ANGPTL3	45.16 (21.90)	51.57 (21.92)	0.001
TNFRI	0.13 (0.76)	0.08 (1.12)	0.051
ADAMTS13	1.15 (0.56)	1.06 (0.56)	0.120
ANGPTL6	37.63 (24.10)	34.15 (20.65)	0.123
ESM-1	617.78 (316.75)	573.31 (267.26)	0.137
Tpo	1.48 (629.54)	1.57 (745.21)	0.176
SCF	0.08 (0.92)	0.05 (1.04)	0.200
CXCL1	228.39 (121.14)	248.14 (170.30)	0.214
TSG-14	1.27 (578.81)	1.35 (658.60)	0.215
PBEF/Visfatin	37.75 (96.07)	28.35 (75.25)	0.271
ICAM-1	803.66 (684.33)	740.51 (648.82)	0.359
uPAR	1.81 (1.07)	1.91 (979.20)	0.376
BMP-2	41.47 (13.65)	40.17 (21.32)	0.505
Angiopoietin-1	0.04 (0.95)	0.02 (1.03)	0.554
Furin	2.35 (1.16)	2.40 (1.12)	0.653
PCSK9	153.75 (261.48)	177.33 (606.84)	0.658
KIM-1	0.02 (0.93)	0.01 (1.04)	0.736
Endoglin/CD105	3.21 (2.53)	3.13 (2.47)	0.769
CD62P	0.01 (1.21)	0.00 (0.85)	0.919
Tie-2	16.31 (8.83)	16.40 (8.94)	0.929

Protein concentrations reported in pg/mL. Bolded *p*-value represents statistical significance (*p* < 0.05). Abbreviations: angiopoietin-like protein (ANGPTL), tumor necrosis factor receptor I (TNFRI), a disintegrin-like metalloproteinase with thrombospondin motif type 1 member 13 (ADAMTS13), endothelial cell-specific molecule-1 (ESM-1), thyroid peroxidase (Tpo), Skp, Cullin, F-box containing complex (SCF), CXC motif chemokine ligand 1 (CXCL1), tumor necrosis factor inducible gene 14 (TSG-14), pre-B cell colony-enhancing factor (PBEF), intercellular adhesion molecule-1 (ICAM-1), urokinase-type plasminogen activator receptor (uPAR), bone morphogenetic protein 2 (BMP-2), Proprotein Convertase Subtilisin/Kexin Type 9 (PCSK9), kidney injury molecule-1 (KIM-1), cluster of differentiation 105 (CD105), P-selectin (CD62P), MACE (major adverse cardiovascular event).

**Table 4:** Adjusted hazard ratios for associations between angiogenesis-related proteins and 2-year major adverse cardiovascular events in patients with peripheral artery disease.

Protein	Hazard Ratio *	95% CI Lower	95% CI Upper	*p*-Value
Endostatin	1.39	1.12	1.71	<0.001
ANGPTL4	1.35	1.08	1.68	<0.001
ANGPTL3	1.35	1.12	1.63	<0.001

* Adjusted for age, sex, hypertension, dyslipidemia, diabetes, past/current smoking, congestive heart failure, coronary artery disease, and previous stroke. Abbreviations: angiopoietin-like protein (ANGPTL), CI (confidence interval).

**Table 5:** Adjusted hazard ratios for associations between angiogenesis-related proteins and 2-year major adverse cardiovascular events in patients without peripheral artery disease.

	Hazard Ratio *	95% CI Lower	95% CI Upper	*p*-Value
Endostatin	1.30	0.74	2.28	0.36
ANGPTL4	1.07	0.67	1.69	0.79
ANGPTL3	1.36	0.94	1.96	0.11

* Adjusted for age, sex, hypertension, dyslipidemia, diabetes, past/current smoking, congestive heart failure, coronary artery disease, and previous stroke. Abbreviations: angiopoietin-like protein (ANGPTL), CI (confidence interval).

## Data Availability

The original contributions presented in the study are included in the article; further inquiries can be directed to the corresponding author.
